# A statistical model for early estimation of the prevalence and severity of an epidemic or pandemic from simple tests for infection confirmation

**DOI:** 10.1371/journal.pone.0280874

**Published:** 2023-01-26

**Authors:** Yuval Shahar, Osnat Mokryn

**Affiliations:** 1 Department of Software and Information Systems Engineering, Ben Gurion University; Beer Sheva, Israel; 2 Department of Information Systems, University of Haifa; Haifa, Israel; University of Toronto, CANADA

## Abstract

Epidemics and pandemics require an early estimate of the cumulative infection prevalence, sometimes referred to as the infection "Iceberg," whose tip are the known cases. Accurate early estimates support better disease monitoring, more accurate estimation of infection fatality rate, and an assessment of the risks from asymptomatic individuals. We find the Pivot group, the population sub-group with the highest probability of being detected and confirmed as positively infected. We differentiate infection susceptibility, assumed to be almost uniform across all population sub-groups at this early stage, from the probability of being confirmed positive. The latter is often related to the likelihood of developing symptoms and complications, which differs between sub-groups (e.g., by age, in the case of the COVID-19 pandemic). A key assumption in our method is the almost-random subgroup infection assumption: The risk of initial infection is either almost uniform across all population sub-groups or not higher in the Pivot sub-group. We then present an algorithm that, using the lift value of the pivot sub-group, finds a lower bound for the cumulative infection prevalence in the population, that is, gives a lower bound on the size of the entire infection "Iceberg." We demonstrate our method by applying it to the case of the COVID-19 pandemic. We use UK and Spain serological surveys of COVID-19 in its first year to demonstrate that the data are consistent with our key assumption, at least for the chosen pivot sub-group. Overall, we applied our methods to nine countries or large regions whose data, mainly during the early COVID-19 pandemic phase, were available: Spain, the UK at two different time points, New York State, New York City, Italy, Norway, Sweden, Belgium, and Israel. We established an estimate of the lower bound of the cumulative infection prevalence for each of them. We have also computed the corresponding upper bounds on the infection fatality rates in each country or region. Using our methodology, we have demonstrated that estimating a lower bound for an epidemic’s infection prevalence at its early phase is feasible and that the assumptions underlying that estimate are valid. Our methodology is especially helpful when serological data are not yet available to gain an initial assessment on the prevalence scale, and more so for pandemics with an asymptomatic transmission, as is the case with Covid-19.

## 1. Introduction

A common problem when attempting to manage epidemics and pandemics at their beginning is assessing the total infection prevalence of the disease. For example, this was often a key issue in the case of the COVID-19 pandemic in its first year. The problem was often referred to colloquially as assessing the total *Infected "Iceberg’s"* size (including the portion of the "Iceberg" that is "underwater," which is composed of asymptomatic infected individuals) [1–5]. Correct estimation of the total infection prevalence also bears directly on the infection fatality rate (IFR); a good lower bound for the first estimate indirectly provides a good upper bound for the second estimate.

One suggestion to solve the problem is to use serological testing of the population, preferably measured randomly, to assess the overall infection prevalence [6–10]. For example, in the case of the COVID-19 pandemic in Spain, using serology has led to a mean of 5% positive seroprevalence using point of care (PoC) testing and a mean of 4.7% positive seroprevalence using a laboratory-based immunoassay testing [11]. In the case of COVID-19 in the UK, a large-scale self-administered immunoassay with over 100,000 volunteers suggested that by the time the serological tests were performed, a mean of 6.4% of the population had been infected [12, 13].

However, serological and antibody home testing often have a known caveat since previously symptomatic people might be more likely to participate in these tests [14]. Another caveat is that in COVID-19, the kinetics of the neutralizing antibody response is typical of an acute viral infection, with declining neutralizing antibody titers observed after an initial peak and a magnitude of this peak that is dependent on disease severity [15]. Furthermore, serological tests are often difficult to administer and costly [16–18].

An alternative strategy for determining infection prevalence is the performance of massive acute disease testing during an epidemic. In the case of the COVID-19 pandemic, one suggested strategy, which attempts to reduce costs, is pooled testing [19]. However, pooled testing requires a dedicated testing infrastructure and overcoming multiple technical hurdles. Other researchers have assessed through simulation the effect of various assumptions on the proportion of asymptomatic cases and their infectivity and compared the results to actual data [20, 21].

Here, we suggest a simple statistical method that uses only the distribution of the data of the patients who are confirmed as positive for the disease in question to set a lower bound on the size of an epidemic’s cumulative infection prevalence (and correspondingly, an upper bound on the IFR). Our method is tailored for the early days of a pandemic, before vaccinations are available, and offers an easy-to-implement tool.

## 2. Methods

We first define our terms and key assumption and then outline our estimation methodology as an algorithm.

### 2.1. Definition and assumptions

Our method for estimating the minimal cumulative infection prevalence relies on finding a sub-group in the population for which the relative risk of being positively confirmed as infected is the highest. We refer to this high-risk sub-group as the *Pivot group*. Since the cumulative infection prevalence was often referred to as the "size of the Iceberg," we define an *Iceberg Factor* (IF) as the ratio of the total size of the infected population to the number of confirmed infected individuals. We further define the *Minimal Iceberg Factor* (MIF) as the smallest IF that explains the number of individuals in the Pivot group.

A key assumption in our method is the almost-random sub-group infection assumption: The overall risk of initial infection, which we refer to as *S*_*0*_, and which is composed of several different components (in particular, the risk of exposure as well as the susceptibility to being infected), is in total (disregarding for a moment the specific values of these components), either almost uniform across all population sub-groups, or at least not higher in the Pivot sub-group; to compute a valid lower bound on the IF, it is enough that *S*_*0*_ should at least not be greater for the Pivot sub-group.

That is, we assume that the initial infection process is a random stochastic process, and thus, the proportion of each infected sub-group within the total infected population is similar to its proportion in the overall population. We demonstrate in the Results Section, using serological data, that the data are consistent with our key assumption, at least for the chosen pivot sub-group. This assumption holds at the early stages of a pandemic before vaccinations are developed and employed. We differentiate the probability of initial infection, *S*_*0*_, from the conditional probability of being symptomatic given that the patient is infected, *S*_*1*_, which is known to be age-related in the case of COVID-19. Thus, even though the initial infection probability *S*_0_ is similar across all sub-groups, such as different age groups. Some sub-groups might well be over- or under-represented within the group of patients confirmed as *positive*. For example, the elderly sub-group might be over-represented in the positively confirmed group of COVID-19 patients despite an almost-uniform *S*_*0*_ because elderly patients have a higher *S*_*1*_, are more likely to be symptomatic after being infected, and thus more likely to be confirmed as positive (The Results support our assumptions).

In this study, we demonstrate our methodology by applying it to the first year of the COVID-19 pandemic and only for demographical sub-groups, specifically age-related subgroups. In general, this focus can be broadened, and other sub-groups, such as those defined by gender or ethnicity, might be used in the analysis. As we shall see when presenting our algorithm, the MIF is, in fact, the Pivot sub-group’s relative risk (*Lift*). Thus, **given the almost-random infection assumption (an almost uniform *S***_***0***_**), this IF is the minimal one that can explain the existence of all of the Pivot sub-group members that were confirmed as positive**.

However, the MIF, and the respective cumulative infection prevalence, might be *smaller* if, by chance, *more* people from the Pivot sub-group within the overall population were "sampled" by the random infection process. Thus, we also need to test whether a sufficiently large number of people from the Pivot sub-group (specifically, the number that is required to explain their existence within the known positively confirmed group) might have been sampled from the overall population in a reasonably likely manner (i.e., in a statistically insignificant fashion), even when given a smaller IF, and thus a smaller overall cumulative infection prevalence.

Therefore, we test the reasonable likelihood of each potential cumulative infection prevalence (corresponding to a given IF) by applying a proportion test. The test examines whether the proportion of the Pivot sub-group in the cumulative infection prevalence of a particular country might be, purely by chance, sufficiently higher than their proportion within the country’s overall population to explain the actual confirmed positive numbers of that Pivot sub-group, but still be larger only in a statistically insignificant fashion. Thus, in our study, in addition to computing the MIF, we calculated the smallest MIF that still explains the number of Pivot Group members that are confirmed as positive but for which the assumption of a "reasonably likely" sampling process due to the infection is not rejected, which we refer to as the *Statistically Insignificant Minimal Iceberg Factor* (**SIMIF**). That is, the SIMIF is the minimal IF for which the proportion test (for the Pivot sub-group’s proportion within the Infected ’Iceberg’) was still insignificant.

### 2.2. An algorithmic description of the method

Our suggested method is as follows:

1. Split the population into disjoint exhaustive sub-groups. For example, by age, gender, or both.
2. Find the *Pivot sub-group*, the population sub-group that displays the maximal relative risk (*Lift*) for being positively confirmed as infected. This is the sub-group for which its proportion within the confirmed (positive) infected patients, compared with its percentage in the population, is the highest.
3. Given the Key Assumption, and thus assuming that the distribution of groups within the infected population is similar to their population distribution, set the MIF to be the Lift of the Pivot sub-group. Thus, the resulting cumulative infection prevalence includes enough members of the Pivot group.
  Note that the *almost*-*random infection assumption* can be relaxed to the assumption that the infection rate of the Pivot sub-group is not greater than that of the rest of the population to maintain the MIF as a lower bound on the IF.
4. To allow for statistical deviations, compute the MIF that, even allowing for an insignificant statistical deviation from the Pivot group’s proportion in the population during the infection process, might still contain a sufficient number of the Pivot group members to explain the number found in the "visible" part of the ’Iceberg’. That is the *Statistically Insignificant Minimal Iceberg Factor* (SIMIF).

Given the MIF, which is a *lower bound* on overall infection prevalence, we compute the *upper bound* on the *Infection Fatality Rate* (IFR), by dividing the number of deaths due to the disease by the size of the estimated Infected "Iceberg" (i.e., the number of positively confirmed cases multiplied by the MIF).

In the Results Section, we demonstrate in detail the application of this method to the COVID-19 pandemic, using data from two countries (the UK and Spain) and then summarize the results for a total of eight different countries; in one of them (the UK) we performed the computation for two data sets acquired at different time points (June and September 2020), and in another case (USA) we performed the calculation for data sets acquired at two different time points from two different regions (New York City and New York State).

Table 1 summarizes each country’s aggregated information of the PCR-RT COVID-19 Confirmed individuals and at which date, the source from which the data was obtained, and the country’s population. We analyzed only secondary data available in the public domain, with no need for approval by the ethics committee in research.

**Table 1:** Covid-19 PCR-RT positive test results data used in this research.

Country	Date	Source	PCR-RT COVID-19 Confirmed individuals	Country population [22]
**Spain**	May 21 2020	Spanish Centro de Coordinación de Alertas y Emergencias Sanitarias [23]	252,283	46,736,782
**UK**	Jun. 10 2020	UK Department of Health and Social Care Statistical Bulletin [24].	222,441	67,530,161
**UK**	Sep. 1 2020	UK Department of Health and Social Care Statistical Bulletin [25].	287,389	67,530,161
**USA NY State**	Mar. 30 2020	US dept. of health [26]	117,522	19,542,209
**USA NYC**	May 16 2020	NYC dept. of Health [26]	357,230	8,336,817
**Italy**	Jun. 24 2020	*Prodotto dall’Istituto Superiore di Sanita (ISS)* [27]	238,042	60,359,546
**Norway**	Jun. 19 2020	The Norwegian Institute of Public Health [28]	8,708	5,378,859
**Sweden**	Dec. 16 2020	Statista, Sweden [29]	357419	10036391
**Belgium**	Jul. 2 2020	Belgian Institute for Health [30]	61,507	11,539,326
**Israel**	Jul. 4 2020	Israel Government Data Repository [31]	28,259	8,519,372

## 3. Results

We first demonstrate our method in detail, using data from Spain and then the UK gathered from the early months of the disease. Then, we consider statistical randomness in the infection probability by computing the interval for the found minimal factor that allows for statistical randomness in the infection within a certain insignificance interval. We then apply our method to nine countries or large regions. We then show that our key assumptions and method are consistent with the serological data for Spain and the UK. We conclude by computing the corresponding Infection Fatality Rate (IFR) for these countries at that time.

### 3.1. Demonstration of the method on early Spanish PCR-RT data

We shall first demonstrate the value and outcomes of our methodology using the COVID-19 PCR-RT data for Spain on May 22, 2020 [23]. At that point, *C*_*pos*_ = 252,283 positive (confirmed) cases were known, as can be seen in Table 2. The table further depicts the number and percent of individuals in each age group out of the country’s population and the number of confirmed (PCR-RT COVID-19 Confirmed individuals) and percent out of all confirmed individuals in each age group.

**Table 2:** Number and age distribution of Spanish and UK citizens within the overall population and within the confirmed COVID-19 cases, for Spain on May 22, 2020, and for the UK on Jun. 10, 2020.

	SPAIN				UK			
Age	# in pop.	% from pop.	# of confirm	% from confirm	# in pop.	% from pop.	# of confirm	% from confirm
0–9	4,340,417	9.29%	998	0.4%	8,065,283	11.95%	1,900	0.85%
10–19	4,682,339	10.02%	1,861	0.74%	7,569,160	11.21%	4,060	1.83%
20–29	4,652,133	9.95%	14,562	5.77%	8,630,614	12.78%	23,728	10.67%
30–39	6,158,281	13.18%	24,075	9.54%	9,203,569	13.63%	27,895	12.54%
40–49	7,935,505	16.98%	36,872	14.62%	8,624,679	12.77%	30,643	13.78%
50–59	6,944,643	14.86%	44,591	17.67%	9,138,365	13.53%	36,343	16.34%
60–69	5,200,462	11.13%	35,713	14.16%	7,206,475	10.67%	23,196	10.43%
70–79	3,921,750	8.39%	33,814	13.4%	5,673,457	8.40%	24,304	10.92%
80+	2,901,252	6.21%	59,797	23.7%	3,418,559	5.06%	50,372	22.64%
All	46,736,782	100%	252,283	100%	67,530,161	100%	222,441	100%

Consider the Spanish age distribution of the confirmed cases. Out of *C*_*pos*_ = 252,283 positive cases, the number of 80 years or older cases, *C*_*pos*.80+_ was 59,797 (23.7%)– 3.82 as much as their proportion in the Spanish population [32], *POP*_*prop*.80+_, which is only 6.21% (2,901,252 of 46,736,782). his sub-group has the highest relative risk (*Lift*) for being confirmed as positive. Thus, the sub-group of 80+ year old people is the Spanish Pivot sub-group, and its Lift is 3.82. Thus, 3.82 would be the MIF for Spain at that point in time.

In other words, at least 963,721 people must have already been infected at that point in time in Spain, to explain the number of positively confirmed cases from its Pivot sub-group.

### 3.2. Demonstration of the method on early UK PCR-RT data

When we follow the same procedure for the United Kingdom using its Jun. 10, 2020 data [24] (see Table 2), the minimal "Iceberg" size that explains the number of positive confirmed cases in the UK’s Pivot, or highest-risk, sub-group, the 80+ years old age-group (4.68% of the British population [33]) at that point in time, *C*_*pos*.80+_ = 50,372, must be at least their relative risk for being confirmed as positive, namely, 4.48 the number of total positive cases found at that time (*C*_*pos*_ = 222,441). Thus, The UK MIF on Jun. 10, 2020, was 4.48.

Therefore, a total of at least *C*_*tot*_ = 1,112,205 British people must have been infected at that point in time, most of them being "underwater" (unconfirmed), to explain the finding at that time of 50,372 positive cases in the 80 + years old age group.

### 3.3. Allowing for statistical deviations

However, based on statistical reasoning, another option might be suggested to explain the number of positively confirmed cases from the Pivot sub-group in Spain or in the UK, using a smaller IF, but without leading to a smaller number of positively confirmed patients from the Pivot sub-group. Perhaps the proportion of infected 80 + years older adults in the cumulative infection prevalence was, by chance, higher than their proportion within the population (even assuming that the likelihood of infection does not depend on age); and somehow, all of the infected older adults were tested and found positive. Could that explain the number of positively confirmed octogenarians while using a smaller IF, namely, a smaller Infected "Iceberg"?

In the case of the Spanish example, note that if the cumulative infection prevalence’s age distribution is similar to that of the Spanish population, it would contain, for an IF of 3.0, only *I*_80+_ = 46,982 cases. Thus, we are short of 12,815 positive patients in that age group. But perhaps the proportion of infected 80 + years older people in the Spanish cumulative infection prevalence is, by chance, higher than their proportion within the Spanish population?

To explore this explanation, we applied a proportion test to see whether it is reasonable that, given the proportion of the 80 + years old population in Spain, enough positive cases might have existed at random within the Spanish cumulative infection prevalence. That is, whether the 2,935,720 people who are 80 + years old, out of Spain’s population of 62,676,180 citizens (i.e., 6.21%), might have randomly produced, through the "random sampling" of being infected, the minimal necessary number of 59,797 positive cases, within an only threefold (i.e., IF = 3.0) ’Iceberg’ size of 756,849 (i.e., 7.9%), assuming an age-oblivious infection process.

The result is: z-statistic = 60.92103; Significance level *p* < 0.0001; 95% CI of observed proportion: 7.84% to 7.96%. (Compare this confidence interval to Spain’s 80 + years age group, which includes only 6.21% of the population). Thus, the Null Hypothesis is rejected at enormous odds. Thus, the IF is highly likely to be larger than three times the total number of confirmed positive cases to explain the number of confirmed cases in the 80+ years age group. In fact, any IF ≤3.76 would result in rejecting the null hypothesis at a level of significance greater than *p* < 0.05. o, the Spanish SIMIF at that point was 3.77.

For the British data and an example factor of four, the results are similar: z-statistic 43.76; Significance level *p* < 0.0001; 95% CI of observed proportion: 5.61% to 5.71%. (Compare this confidence interval to the UK’s 80 + years age group, which includes only 4.68% of the population). Thus, the British IF then must have been larger than four. In fact, for the UK, on Jun. 10, 2020, any IF ≤4.43 would result in rejecting the null hypothesis at a level of significance greater than *p* < 0.05, so the UK SIMIF at that point was 4.44. Since not all infected cases were confirmed as positive, the prevalence of both the Spanish and the UK cumulative infections must have been larger.

We followed this procedure for multiple countries or large regions whose data, mostly during the early COVID-19 pandemic phase, were available, as detailed in Table 1. The results are detailed for all these countries, in detail, in S1 Table in S1 File.

### 3.4. Assessment of the key assumption’s consistency and of the method’s computational results

All that remains now is to assess that our key assumption that the initial infection sub-group infection-susceptibility S_0_ is indeed age-invariant, and in particular, not significantly higher for our Pivot group, which in this case consists of the older people, is consistent with the data. We can easily validate this assumption by examining serological testing results from Spain and the UK (Table 3), depicted by Age group and the type of test. In the Spanish population, blood samples were taken during Apr. 27 to May 11, from 61,075 participants who received a point-of-care antibody test; if they agreed, a more definitive chemiluminescent microparticle immunoassay was also performed. The mean portion of older adults demonstrating evidence for previous COVID-19 infection was quite similar, considering both test types, to the portion of seropositive cases within the other age groups. In the case of the laboratory-based immunoassay, it is even lower than that portion within all other age groups, except for children and adolescents. Serological tests in the UK were performed during Jun. 20 to July 136, using a self-administered lateral flow immunoassay (LFIA) test for IgG among a random population sample of 100,000 adults over 18 years. The results certainly do not suggest a higher infection-susceptibility risk, *S*_0_, for the elderly population: The portion of 75 + years old adults demonstrating evidence in their blood samples for previous COVID-19 infection was the lowest of all age groups for which the test was performed, thus further validating our assumption.

**Table 3:** Serology results for COVID-19 in Spain from Apr. 27 to May 11, 2020, using two different serological tests, and in the United Kingdom from Jun. 20 to Jul. 13, 2020, using lateral flow immunoassay (LFIA). Estimates of prevalence adjusted for imperfect test sensitivity and specificity; a 95% Confidence Interval is specified for each estimate.

SPAIN			UK	
Relative frequency of positives (%)			Relative frequency of positives (%)	
Age	Point-of-care test	Immunoassay	Age	LFIA
0–19	3.4 [2.9–3.9]	3.8 [3.2–4.6]	18–24	7.1 [6.5–7.8]
20–34	4.4 [3.7–5.1]	5.0 [4.3–5.8]	25–34	7.0 [6.5–7.4]
35–49	5.3 [4.7–5.9]	4.9 [4.3–5.5]	35–44	5.7 [5.3–6.0]
50–64	5.8 [5.3–6.5]	4.7 [4.1–5.3]	45–54	6.1 [5.8–6.4]
>64	6.0 [5.4–6.8]	4.5 [3.8–5.3]	55–64	5.5 [5.2–5.9]
			65–74	3.7 [3.4–4.0]
			75+	3.6 [3.2–4.1]
Spain cumulative infection prevalence by serology: 4.58–5.033			UK cumulative infection prevalence by serology: 5.25–5.96	

In both countries, the actual IFs computed from the serological tests (9.32 by PoC or 8.49 by immunoassay for Spain and 17.00 for the UK) were, as predicted, considerably higher than the MIF lower bound computed by our method (3.82 and 4.48) for chronologically similar periods, and certainly higher than the SIMIF. Thus, these results are consistent with our key assumption and are also consistent with our method’s computational results. Based on a Proportion Test, there might be several statistically significant differences in the serological prevalence of the infection among several of the population’s age sub-groups, at least in the case of the UK. However, the Key Assumption, namely that the Pivot sub-group is not infected at a higher rate than the rest of the population, still holds.

We note here that The MIF and the SIMIF were quite close in the cases we analyzed in detail, implying that calculating the MIF is probably sufficient for most cases.

### 3.5. Computing the infection fatality rate (IFR)

We proceed to compute the upper bound on the IFR. Recall that the MIF is a lower bound on the IF. Then, the estimated Infected "Iceberg" size is the number of positively confirmed cases multiplied by the MIF.

We can compute an upper bound on the IFR by dividing the size of the estimated cumulative infection prevalence by the fatalities from the infection. Given the COVID-19 disease data, we chose the fatality rate date to be two weeks later than the date of the Serology test.

The IFR upper bound computed by the lower bound provided by the MIF in Spain (given the number of deaths by Jun. 6, 2020, two weeks after May 22, 2020) was 3.03%, while the IFR computed by serology test results (which can be considered as closer to the true IFR) was 1.24% (PoC) or 1.36% (immunoassay); for the UK, the IFR upper bound computed by the lower bound provided by the MIF was 4.04%, while the IFR computed by serology was 1.06%; for New York State, the IFR upper bound computed by the lower bound supplied by the respective MIF was 9.29%, while IFR calculated from the serological test results was 0.59%. The full details appear in Table 4, showing for countries for which we have serological data, the date at which the serology data was reported and the serology test type, the COVID-19 fatality rate date, and the corresponding death toll at that date, The Serology-based IF and cumulative infection prevalence, and the calculated IFR according to the Serology and according to the MIF.

**Table 4:** Upper bounds on the infection fatality rate (IFR) of covid-19 for countries in which serological test results were available, calculated through the minimal iceberg factor (MIF) and through the actual serological test results.

Country	Serology type	Serology date	Covid-19 death toll [34]	Covid-19 death toll date	Serology-based Iceberg Factor	Serology-based Iceberg Size	Serology-based IFR %	MIF_based Upper bound on IFR %
Spain	PoC	5.11.2020	29204	6.6.2020	9.32	2,352,262	1.24	3.03
Spain	Immunoassay	5.11.2020	29204	6.6.2020	8.49	2,141,993	1.36	3.03
UK June	LFIA	7.13.2020	41167	7.27.2020	17.00	3,781689	1.06	4.04
NYS US	Immunoassay	3.30.2020	15500	4.15.2020	22.23	2,612,268	0.59	9.29

## 4. Discussion

Estimating a lower bound for the total number of infected individuals in a given population is key to monitoring and managing a new epidemic, and certainly a new pandemic, in its early days. It is also beneficial when a new strain of an existent virus appears. Along with other benefits, it supports an assessment of the risk due to asymptomatic cases and the creation of a more realistic upper bound on the IFR, which is important for quick evaluation of the risk to the population from a new epidemic or pandemic.

Here, we suggest a cheap and easy method for rapid estimation of this lower bound, using data from the readily available tests taken by the public (in the case of COVID-19, this is the PCR-RT test). The line of reasoning of our method is based on finding the highest-lift Pivot group for being infected, that is, the sub-group for which its proportion within the confirmed (positive) infected patients, compared with its percentage in the population, is the highest. Our algorithm then utilizes the found Lift to determine the minimal factor which, when multiplied by the Pivot group size, will yield an estimate of the cumulative infection prevalence and show that this estimate is a lower bound on the cumulative infection prevalence.

A key assumption in our method, which we had demonstrated as consistent with the serological data in the countries we had analyzed, is the *almost*-*random* sub-group *infection assumption*: The risk of initial infection is either almost uniform across all population sub-groups or not higher in the Pivot sub-group. We differentiate here the susceptibility to *infection*, S_0_, assumed to be almost-randomly uniform across all population sub-groups at this early stage, from the probability of *developing symptoms and complications*, and hence being confirmed as positive, S_1_, which differs between sub-groups (e.g., by age).

We also explicitly consider statistical randomness in the infection probability by computing the interval for the found minimal factor that allows for statistical randomness in the infection within a certain insignificance interval. Our results suggest that calculating the minimal factor, a quick and easy calculation, is probably sufficient.

We have demonstrated the consistency of our assumption and the validity of our method using UK and Spain serological surveys of COVID-19 in its first year. Our key assumption was consistent with the data of the age-related pivot groups: The immunoassay results show that in Spain, the cumulative infection rate for ages 65+ was the lowest other than for the youngest group and that in the UK, the cumulative infection rate was actually lowest for the older population groups.

Furthermore, the serology-based infection-prevalence factors computed from the Spanish and UK-based data were consistent with our lower-bound MIF predictions.

Overall, we applied our methods to nine countries or large regions whose data, mostly during the early COVID-19 pandemic phase, were available: Spain, the UK at two different time points, New York State, New York City, Italy, Norway, Sweden, Belgium, and Israel. For each, we established the minimal lower bound on the factor by which the number of positively confirmed patients should be multiplied, which explains the population’s age-based distribution, assuming an infection susceptibility that is independent of age. The lower bound ranged from 1.35 (NYC) to 5.1 (Belgium). We have also computed the corresponding IFRs for each country or region.

The most prevalent solution for assessing the cumulative infection prevalence in the population is the use of serological or home antibody testing of the population, preferably measured randomly [35, 36]. They enable detection only a few weeks after the infection, as antibodies are produced only 1–2 weeks after the onset of the infection, and require trained laboratory staff [37]. Thus, unlike the COVID-19 PCR-RT tests, which were administered in vast numbers, serology tests were performed on a much more limited scale [7].

Comparing the results of our method to serological data of a similar period in Spain and the UK, we find that the results from the serological data were 2.2 and 3.7 times higher, respectively, than our estimation of a lower bound for the epidemic’s infection prevalence in these areas. While the antibody tests give a more accurate estimate, we believe that gaining an insight into the prevalence of a disease in the presence of asymptomatic individuals that spread the disease is important by itself. There are several advantages to using our method with the PCR-RT tests in the case of COVID-19. The common nucleic acid-based method in its base is highly accurate and readily available and allows for high throughput through simple automation services while returning the results in a matter of a few hours. Thus, it has been administered in very large numbers in almost all countries, the results are readily available, and due to the large number of administered tests, randomness is easily assumed [7, 38, 39]. Longitudinal PCR-RT tests were used for evaluating the percentage of asymptomatic people among all tested, based on self-reports of lack of symptoms [20, 40–42]. Compared with our method, longitudinal test results require monitoring a specific region over time and thus cannot be used at large. Thus, our method is the only one to use readily available information from large-scale PCR-RT tests and infer a realistic lower bound on the prevalence of the disease in the population.

Unlike alternative strategies for determining infection prevalence, which advocates the performance of massive acute disease testing during an epidemic or a pandemic, such as pooled testing [19], our simple methodology requires no dedicated testing infrastructure and does not necessitate overcoming multiple technical hurdles. Our method, which is based on the actual tests being performed, and which provides lower bounds, is also simpler and arguably more sound than approaches using simulation [20, 21].

Our method requires identifying a Pivot group. We use, for COVID-19, the age sub-groups. This seems to have worked reasonably well in the cases that we had analyzed, but other options are quite possible. For example, in the case of New York City, our computed MIF (1.35) is much smaller than the serology-based one (22.23); although the result is still consistent with the lower bound, the extreme gap in value might be due to the fact that in New York City a better Pivot group might have been based on socio-economic and ethnic considerations; these factors have been shown to play a key role in the prediction of morbidity and especially mortality across different counties on the USA [43]. Furthermore, the MIF is only a *lower bound*: In practice, only a portion of the Pivot sub-group’s infected members are likely to be confirmed as positive.

There are several limitations to our methodology. In particular, it is useful when positively confirmed cases are detected mostly due to a symptomatic presentation by the patients (which is governed by the symptom-manifestation probability S_1_), as was common during the early phase of the COVID-19 pandemic or when some underlying process creates a high variability between different sub-groups, regarding the probability of being positively confirmed. It is less useful when positively confirmed cases are detected at random, such as when a general screening of the population is performed (whose results are governed by the infection-susceptibility probability *S*_*0*_). The latter situation became more common during the more advanced phases of the COVID-19 pandemic, as the number of tests grew and the indications for performing them had expanded.

Often, it will be easy to determine an appropriate Pivot sub-group (based on gender, age, or ethnicity). But, in other cases, it might be more challenging to do that. However, even in a new outbreak whose characteristics are relatively unknown, as long as one manages to find a population sub-group whose infection rate seems to be not higher than that of the other sub-groups, but its confirmed-positive rate (presumably due to a higher symptomatic presentation rate) seems to be higher than most other sub-groups, it could be used as a useful Pivot sub-group for calculating a lower bound for the prevalence of the overall infection. Furthermore, judging from outbreaks of the past several decades, it usually does not take too long to find a reasonable Pivot sub-group. For example, in the case of the SARS-CoV-2 outbreak, it was clear after only a few months that one such subgroup is the elderly population; in the case of the USA, it seemed to include also certain ethnic and socio-economic sub-groups, as documented in several studies; in other outbreaks it might be, for example, diabetic patients, etc.

We ignored, in the case of the COVID-19 pandemic, the PCR-RT sensitivity and specificity; we assume they do not vary across age groups. Note also that the number of confirmed cases is by itself only a lower bound due to the PCR-RT’s limited sensitivity.

### 4.1. Conclusions

We have demonstrated that estimating a lower bound for an epidemic or pandemic’s infection prevalence and an upper bound on its infection fatality rate at its early phase, using our methodology, is feasible, and that the assumptions underlying that estimate are consistent with the data.

Our methodology might often be necessary for the early phases of an epidemic or pandemic when serological data are not yet available, when vaccines are not available, or when new mutations of a known virus appear, which are resistant to an existent vaccine.

Computing The MIF might also add insights to pandemic-related differences across different countries and times.

## Supporting information

S1 File (DOCX)
