# Supplementary material for: A statistical model for early estimation of the prevalence and severity of an epidemic or pandemic from simple tests for infection confirmation
Source: PLoS One. 2023 Jan 26;18(1):e0280874. doi: 10.1371/journal.pone.0280874 (PMC9879391; doi:10.1371/journal.pone.0280874)
Supplement: S1 File — (DOCX) [file pone.0280874.s001.docx]

Supplementary Material for the paper:
A Statistical Model for Early Estimation of
the Prevalence and Severity of an Epidemic or Pandemic
 from Simple Tests for Infection Confirmation

Yuval Shahar^1^, Osnat Mokryn^2^*

^1^Department of Software and Information Systems Engineering, Ben Gurion University; Beer Sheva 84105, Israel.  Email: [yshahar@bgu.ac.il](mailto:yshahar@bgu.ac.il).

^2^Department of Information Systems, University of Haifa; Haifa 34988, Israel.  Email: omokryn@is.haifa.ac.il.
* Corresponding author

**Supplementary Results: Applying the method to additional countries and regions**

We followed this procedure for multiple countries or large regions whose data, mostly during the early COVID-19 pandemic phase, were available, as described in the main paper Table S1 (Spain (1), the UK at two different time points (2,3), New York State (USA) (4), New York City (USA) (5), Italy (6), Norway (7), Sweden (8), Belgium (9), and Israel (10)). For each of them, we established the minimal lower bound on the IF that explains the population age-based distribution, assuming an age-independent S_0_. The lower bound ranged from 1.35 (NYC) to 5.1 (Belgium). The results are summarized in Table S1, showing for each country the date the data was collected; the number of individuals that were PCR-RT positive termed Tested Positive; the age group of the Pivot group for which the relative risk is higher, their percentage in the population and their relative risk. Then we show the MIF calculated from the Pivot group information and the ‘Iceberg’ size that corresponds to the MIF, for each country. We continue to show the result of the numerical calculations by depicting the SIMIF and its corresponding ‘Iceberg’ size, and the results of the Proportion test for the SIMIF for the Pivot group (z-stat, p-value, and confidence interval). The last column shows for each country and data date the Interval within which the SIMIF becomes significant.

Table S1 Minimal Iceberg Factor (MIF), Statistically Insignificant Minimal Iceberg Factor (SIMIF), and proportion test calculations for the COVID-19 pandemic Pivot groups in eight different countries, for ten different dates

| **Country** | | **Spain** | **USA NYS** | **USA NYC** | **UK** | **UK** | **Italy** | **Norway** | **Sweden** | **Belgium** | **Israel** |
| --- | --- | --- | --- | --- | --- | --- | --- | --- | --- | --- | --- |
| **MM.DD.YY** | | **5.22.20** | **3.31.20** | **5.16.20** | **6.10.20** | **9.1.20** | **6.24.20** | **6.19.20** | **12.16.20** | **7.2.20** | **7.4.20** |
| Tested Positive | | 252,283 | 117,522 | 357,230 | 222,441 | 287,389 | 238,042 | 8,708 | 357419 | 61,507 | 28,259 |
| Pivot group(Covid-19 age group) | | 80+ | 50-64 | 55-64 | 80+ | 80+ | 80+ | 50-59 | 40-49 | 80+ | 20-29 |
| Pivot % of pop. | | 6.21% | 20.01% | 11.98% | 5.06% | 5.06% | 7.17% | 13.04% | 12.73% | 5.71% | 13.84% |
| relative Risk of Pivot for pos. | | 381.83% | 141.31% | 134.54% | 447.33% | 379.74% | 353.21% | 142.39% | 142.44% | 509.98% | 158.81% |
| MIF | MIF | 3.82 | 1.42 | 1.35 | 4.47 | 3.8 | 3.55 | 1.43 | 1.43 | 5.1 | 1.6 |
|  | Total Implied Iceberg size | 963,721 | 166,881 | 482,261 | 996,536 | 1,092,078 | 845,049 | 12,452 | 511,109 | 313,686 | 45,214 |
| SIMIF Proportion Test | SIMIF | 3.76 | 1.39 | 1.33 | 4.43 | 3.76 | 3.5 | 1.34 | 1.41 | 5.02 | 1.6 |
|  | Total Implied Iceberg size | 953,630 | 165,356 | 475,115 | 985,414 | 1,080,583 | 833,147 | 11,669 | 503,961 | 308,765 | 43,801 |
|  | Pivot proportion from SIMIF Iceberg | 6.27% | 20.34% | 12.12% | 5.11% | 5.11% | 7.24% | 13.86% | 12.86% | 5.80% | 14.18% |
|  | z-stat | 2.428 | 3.334 | 2.972 | 2.265 | 2.371 | 2.477 | 2.63 | 2.769 | 2.155 | 2.061 |
|  | p-value | 0.0152 | 0.0009 | 0.003 | 0.0235 | 0.0177 | 0.0133 | 0.0085 | 0.0056 | 0.0311 | 0.0393 |
|  | 95% CI | 6.22%-6.32% | 20.15%-20.541% | 12.03%-12.21% | 5.07%-5.15% | 5.07%-5.15% | 7.18%-7.3% | 13.24%-14.5% | 12.77%-12.95% | 5.72%-5.88% | 13.83%-13.84% |
| Interval within which SIMIF becomes significant | | [3.76,3.77] | [1.39,1.40] | [1.33,1.34] | [4.43,4.44] | [3.76,377] | [3.5,3.51] | [1.34-1.35] | [1.41-1.42] | [5.02-5.03] | [1.55-1.56] |

**References**

1. De C de C, Emergencias A y, Sanitarias. Actualización n^o^ 113. Enfermedad por el coronavirus (COVID-19). 22.05.2020 SITUACIÓN EN ESPAÑA.

2. Public Health England. The weekly surveillance report in England: Week 04 June 2020 to 10 June 2020. 2020.

3. Public Health England. The weekly surveillance report in England: Week 26 August 2020 to 01 September 2020. 2020.

4. The US Department of Health & Human Services. New-York COVID-19 Reported Patient Impact and Hospital Capacity by Facility [Internet]. 2020 [cited 2020 Apr 21]. Available from: https://healthdata.gov/Hospital/COVID-19-Reported-Patient-Impact-and-Hospital-Capa/anag-cw7u

5. NYC health department Coronavirus data Github [Internet]. [cited 2020 Dec 29]. Available from: https://github.com/nychealth/coronavirus-data/blob/master/totals/by-age.csv

6. Riccardo F, Andrianou X, Bella A, Del Manso M, Mateo Urdiales A, Fabiani M, et al. Prodotto dall’Istituto Superiore di Sanità (ISS), Roma A cura di. 2020.

7. The Norwegian Institute of Public Health. Norway Covid Cases June 2020.

8. Statista. Sweden: coronavirus cases by age, accessed Dec. 16th 2020 [Internet]. 2020 [cited 2021 Mar 2]. Available from: https://www.statista.com/statistics/1107905/number-of-coronavirus-cases-in-sweden-by-age-groups/

9. The Belgian institute for health Sciensano. Epistat – COVID-19 Belgian Dashboard [Internet]. [cited 2021 Mar 2]. Available from: https://epistat.wiv-isp.be/Covid/covid-19.html

10. National Digital Department. COVID-19 Israeli Government Data Repository: Corona cases grouped by age [Internet]. [cited 2021 Mar 2]. Available from: https://data.gov.il/dataset/covid-19
